# Case Report: Hemophagocytic lymphohistiocytosis masquerading as drug-induced liver injury: successful treatment with low-dose ruxolitinib and glucocorticoids

**DOI:** 10.3389/fimmu.2026.1856139

**Published:** 2026-05-28

**Authors:** Yang Dai, Luocheng Zhang, Xushu Zhong, Ailin Zhao, Ting Niu

**Affiliations:** 1 Department of Hematology, Institute of Hematology, and Center for High Altitude Medicine, West China Hospital, Sichuan University, Chengdu, China; 2 State Key Laboratory of Biotherapy, Collaborative Innovation Center of Biotherapy, West China Hospital, Sichuan University, Chengdu, China; 3 National Facility for Translational Medicine (Sichuan), West China Hospital, Sichuan University, Chengdu, China; 4 West China Medical School, West China Hospital, Sichuan University, Chengdu, China

**Keywords:** corticosteroids, Cytokine storm, DILI (drug induced liver injury), HLH, hemophagocytic lymphohistiocytosis, Ruxolitinib

## Abstract

**Background:**

Hemophagocytic lymphohistiocytosis (HLH) is a life-threatening hyperinflammatory syndrome that can present with drug-induced liver injury (DILI), often mimicking acute liver failure and delaying diagnosis. Therapeutic options are limited in patients with severe hepatic dysfunction.

**Objectives:**

To describe the clinical features, treatment, and outcomes of three adult patients presenting with DILI-mimicking HLH and evaluate the effectiveness of low-dose ruxolitinib combined with corticosteroids.

**Methods:**

Three patients with DILI-like hepatic injury and secondary HLH were retrospectively analyzed. All patients exhibited severe hyperbilirubinemia and cytopenias refractory to conventional supportive therapy. Low-dose ruxolitinib plus corticosteroids were administered. Laboratory, imaging, genetic, and histopathological data were reviewed.

**Results:**

All patients responded rapidly to the combination therapy, with progressive normalization of bilirubin and inflammatory markers. Transient declines in blood counts occurred but resolved without intervention. Genetic testing revealed heterozygous variants in immune-regulatory genes (NLRP3, DOCK8, MYO5A, PLCG2) classified as variants of uncertain significance, suggesting a potential predisposing role. No significant adverse events related to ruxolitinib were observed.

**Conclusions:**

HLH can initially present as severe DILI, posing diagnostic challenges. Short-course, low-dose ruxolitinib combined with corticosteroids is a safe and effective treatment in patients with hepatic-predominant HLH. Early recognition and timely immunomodulatory therapy may improve outcomes in this life-threatening but treatable condition.

## Introduction

Hemophagocytic lymphohistiocytosis (HLH) is a life-threatening hyperinflammatory syndrome characterized by uncontrolled activation of macrophages and cytotoxic T cells, leading to excessive cytokine release and multiorgan dysfunction (1). HLH is broadly classified into primary (genetic) and secondary forms. Secondary HLH is more common in adults and is typically triggered by infections, malignancies, autoimmune diseases, and, increasingly recognized, medications (2, 3). Recent evidence, including large adult HLH cohorts, has highlighted that drug exposure represents an important but underappreciated cause of secondary HLH, likely mediated through immune dysregulation and aberrant cytokine activation (4).

Drug-induced hypersensitivity reactions, particularly drug-induced hypersensitivity syndrome (DIHS) or drug reaction with eosinophilia and systemic symptoms (DRESS), have been increasingly implicated in the development of secondary HLH. These conditions share overlapping clinical and immunological features, including fever, cytopenia, rash, and multiorgan involvement, suggesting a continuum of immune dysregulation (5). In this context, liver involvement is common and may range from mild transaminitis to severe drug-induced liver injury (DILI), and in some cases, acute liver failure. However, the recognition of HLH in patients presenting with predominant hepatic manifestations remains challenging, as clinical features may initially mimic isolated DILI or acute liver failure, leading to delayed diagnosis and treatment (6).

In this study, we report a case series of three patients who initially presented with severe drug-induced liver injury and were subsequently diagnosed with secondary HLH. All patients demonstrated rapid clinical response to low-dose ruxolitinib combined with corticosteroids. This study was conducted in accordance with the Declaration of Helsinki and approved by the Institutional Review Board of West China Hospital, Sichuan University (Approval No. WCH-EC/2021-1621). Through detailed clinical characterization and a review of previously reported cases, we aim to highlight DILI as an underrecognized trigger of HLH and to explore the potential role of JAK-STAT inhibition as a liver-sparing therapeutic strategy in this setting.

## Case presentation

### Case 1

A 43-year-old woman was admitted to our institute on October 24, 2025, with a 1-month history of skin rash and 20 days of fever, abdominal distension, and anorexia.

**Figure 1:**
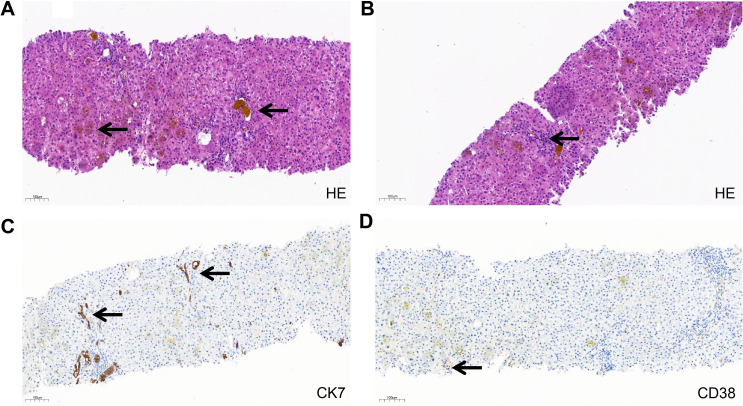
Pathological features of liver tissue in case 1. **(A, B)** Hematoxylin and eosin (H&E) staining of liver biopsies from Case 1 **(A)** and Case 2 **(B)**, showing hepatocyte swelling, focal necrosis, and bile thrombi (arrows). **(C)** CK7 immunohistochemistry highlighting bile ductular proliferation in Case 1 (arrows). **(D)** CD38 immunohistochemistry showing plasma cell infiltration in Case 2 (arrow). Scale bars = 100 μm in all panels; note that the scale bars are relatively small relative to the field of view.

One month ago, the patient developed pruritic rashes involving the perianal region, hands, and feet. Oral and intramuscular medications (not specified) from a local hospital improved cutaneous symptoms soon. Twenty days ago, she developed a fever with a highest temperature of 38.4 °C. Following administration of a single dose of unspecified traditional Chinese medication from a local clinic, she experienced progressive abdominal distension and anorexia, accompanied by fatigue, productive cough, and dyspnea. Laboratory tests from a local hospital revealed liver dysfunction: total bilirubin (TB) 72.3 μmol/L, direct bilirubin (DB) 50.0 μmol/L, alanine aminotransferase (ALT) 1,746 U/L, and aspartate aminotransferase (AST) 1,222 U/L. Despite treatment with hepatoprotective agents, choleretic therapy, and antibiotics, jaundice and constitutional symptoms worsened. Her bilirubin levels rose progressively, and high-grade fever persisted (maximum 40 °C). The patient was subsequently transferred to our institute and was admitted to the infectious disease department for a diagnosis of liver failure. On admission, she was noticeably jaundiced; the remainder of the physical examination was unremarkable. Laboratory tests showed: Complete blood count (CBC): hemoglobin 107 g/L (115–150 g/L), platelets 120 × 10^9^/L (100–300 × 10^9^/L), white blood cell count 5.05 × 10^9^/L (3.5–9.5 × 10^9^/L), neutrophils 3.64 × 10^9^/L (1.8–6.3 × 10^9^/L), TB 266.9 μmol/L (≤23.0 μmol/L), DB 199.6 μmol/L (≤6.8 μmol/L), ALT 280 U/L (9–50 U/L), AST 706 U/L (15–40 U/L), alkaline phosphatase 110 U/L (45–125 U/L), γ-glutamyl transferase 106 U/L (10–60 U/L), albumin 24.2 g/L (40.0–55.0 g/L); triglycerides 2.05 mmol/L (0.29–1.7); ammonia 106.2 μmol/L (11.0–51.0 μmol/L), prothrombin time (PT) 32.6 seconds (9.6–12.8 seconds), Activated Partial Thromboplastin Time (APTT) 60.8 seconds (24.8–33.8 seconds), Thrombin Time (TT) 27.1 seconds (14.0–22.0 seconds), Fibrinogen 0.78 g/L (2.0–4.0 g/L), Serological testing for hepatitis A, B, C, and E viruses were all negative, cytomegalovirus (CMV) DNA: <50.00 copies/mL. Epstein-Barr virus (EBV) DNA: <50.00 copies/mL. Serology for rubella virus, Toxoplasma gondii, herpes simplex virus type I, and type II showed positive IgG with negative IgM, indicating past infection rather than acute infection. Ferritin 331 ng/mL (13–150), soluble interleukin-2 receptor (sCD25) 2971 U/mL (223–710). Abdominal CT scan demonstrated heterogeneous decreased hepatic parenchymal density with reduced liver volume and lymphatic stasis pattern, splenomegaly. Upper abdominal MRI revealed mild hepatomegaly with multiple patchy areas showing hypointense T1 and mildly hyperintense T2 signals, predominantly distributed in the right hepatic lobe. These lesions demonstrated no restricted diffusion, heterogeneous enhancement, and scattered patchy delayed enhancement. Associated findings included mild narrowing and blurring of the hepatic veins, with luminal narrowing of the intrahepatic inferior vena cava segment. The portal vein was not dilated, with mildly widened and blurred perivascular spaces. Mild splenomegaly was noted without parenchymal abnormality. No lymphadenopathy was identified in the upper abdomen or retroperitoneum. Thickening of the mesentery, omentum, bilateral perirenal fascia, and parietal peritoneum was observed, with scattered fluid collection in the upper abdominal cavity. Positron emission tomography/computed tomography (PET/CT) demonstrated no definitive evidence of malignancy throughout the body. The liver showed heterogeneous decreased attenuation with mildly reduced glucose metabolism, suggestive of hepatic injury. Liver biopsy was performed, which showed approximately 18 portal tracts were examined. Hepatocyte swelling with focal ballooning degeneration was observed. No definite steatosis was identified. Hepatocyte regeneration with rosette formation was present. Scattered, spotty necrosis was noted within the lobules. Sinusoidal dilatation was not prominent. Cholestasis with bile thrombus formation was seen in some hepatocytes. Mild interface hepatitis was present. Portal tracts showed mild to moderate infiltration of lymphocytes, mononuclear cells, scattered plasma cells, and neutrophils. Interlobular bile ducts were not significantly reduced; however, prominent bile ductular proliferation was noted around the periphery. Foot and Masson stains demonstrated fibrous tissue proliferation with portal tract expansion and fibrous septa formation (**Figure 1**). Given the rapid onset of liver injury following previous drug exposure, DILI was suspected, and artificial liver support therapy was initiated. Following the therapy, bilirubin levels decreased significantly but rose again shortly thereafter. Moreover, the fever persisted. The patient’s persistent fever lacking overt infectious features prompted suspicion of HLH. Hematology consultation was obtained, and the patient was subsequently transferred to our department for specialized management. Thereafter, a comprehensive workup for HLH was performed, which included: autoimmune antibody screening was negative; bone marrow smear showed hemophagocytosis, otherwise unremarkable; flow cytometry revealed no distinct abnormal immunophenotypic cells. Bone marrow biopsy showed no significant abnormalities. Based on the fever, splenomegaly, low fibrinogen level, elevated sCD25 and hemophagocytosis (in bone marrow), fulfilling 5 of 8 HLH-2004 diagnostic criteria and establishing a diagnosis of HLH. Although etoposide plus dexamethasone therapy (ED regimen) remains the standard treatment for HLH, given the patient’s severe hepatic impairment, etoposide might further exacerbate liver injury. Given the increasing evidence for ruxolitinib use in HLH and following a comprehensive discussion with the patient and her family, low-dose ruxolitinib (5 mg twice daily) combined with intravenous dexamethasone (10 mg once daily) was initiated after obtaining informed consent. Genetic screening for HLH-associated mutations was performed as well, which yielded a heterozygous NLRP3 mutation (c.1371G>T [p.E457D]). With the treatment, the patient’s bilirubin levels decreased rapidly, and HLH-related parameters also showed significant improvement. The patient was discharged 28 days later. Pre-discharge laboratory tests showed complete blood count with Hb 82 g/L and normal white blood cell and platelet counts. Liver function tests revealed TB 116.1 μmol/L, DB 92.2 μmol/L, ferritin 403 ng/mL, with normalized sCD25. The patient continued oral ruxolitinib 5 mg twice daily for one month, with dexamethasone tapered and discontinued over two months. The patient has remained afebrile with normal liver function during 4 months of follow-up, with entirely normal complete blood count and liver function at the last visit on April 2, 2026.

### Case 2

A 21-year-old woman was admitted on March 5, 2026, with a 3-month history of multiple lymphadenopathies accompanied by fever. Three months prior to admission, the patient palpated bilateral axillary and left cervical lymphadenopathy, approximately 2 cm in diameter, followed by recurrent fever with a maximal temperature of 39.6 °C, accompanied by chills, diaphoresis, and fatigue. No headache, dizziness, nausea, vomiting, sore throat, myalgia, arthralgia, or jaundice was reported. Fever initially responded to oral ibuprofen but later recurred. The patient went to a local hospital and underwent a left inguinal lymph node biopsy, which demonstrated lymphoid follicular hyperplasia with expanded germinal centers, consistent with reactive lymphoid hyperplasia. Laboratory tests showed: ALT 162 U/L, AST 272 U/L; TB and DB values were not available. There, she was given antibiotic therapy, hepatoprotection, and antipyretic treatment, but the symptoms persisted without significant amelioration. Six days prior to admission, a generalized erythematous rash developed, predominantly involving the lumbosacral region. The patient presented to our institute. On admission, the patient was markedly agitated and noticeably jaundiced; the liver, spleen, and lymph nodes were not palpable. Laboratory tests revealed: hemoglobin 80 g/L, platelet count 43 × 10^9^/L, white blood cell count 1.03 × 10^9^/L, absolute neutrophil count 0.77 × 10^9^/L; PT 19.0 seconds, international normalized ratio (INR) 1.72, APTT 47.4 seconds, TT 47.4 seconds, fibrinogen 0.80 g/L; TB 238.9 μmol/L, DB 183.1 μmol/L, total bile acids 308.8 μmol/L, ALT 470 U/L, AST 1,159 U/L, alkaline phosphatase 108 U/L, γ-glutamyl transferase 77 U/L, total protein 52.5 g/L, albumin 31.1 g/L, triglycerides 3.13 mmol/L, lactate dehydrogenase (LDH) 1,098 U/L (120–250 U/L); ferritin 45,174 ng/mL (30–400 ng/mL). sCD25 5,182 U/mL (223–710 U/mL). Serologic markers for hepatitis A, B, C, and E viruses were all non-reactive; both CMV DNA and EBV DNA were below the lower limit of detection. Comprehensive autoimmune serologic screening revealed no detectable autoantibodies. Chest and abdominal CT scan revealed: hepatosplenomegaly with heterogeneous decreased hepatic attenuation, hepatic injury or other etiology to be correlated with liver function; scattered linear exudative changes in perihepatic spaces. The patient presented with fever persisting over one week, splenomegaly, pancytopenia, low fibrinogen level/elevated triglyceride, markedly elevated ferritin and sCD25 levels, establishing the diagnosis of HLH. Genetic screening for HLH-associated mutations revealed a heterozygous MYO5A mutation (c.3489 + 7G>T) and a heterozygous DOCK8 (c.986C>T [p.A329V]). In view of the patient’s severe agitation, comprehensive communication with the family was undertaken and informed consent was obtained. Low-dose ruxolitinib (5 mg twice daily) plus dexamethasone 10 mg intravenously once daily was then initiated. Additionally, the patient was treated with magnesium isoglycyrrhizinate and polyene phosphatidylcholine for hepatoprotection. In parallel, a comprehensive workup to identify the cause of HLH was performed: bone marrow aspirate smears, flow cytometry, and trephine biopsy all showed no significant abnormalities. PET/CT demonstrated hepatosplenomegaly without significantly increased metabolic activity. No other abnormalities were identified. Liver biopsy was recommended, but the patient declined. After 8 days of treatment, liver function, hemophagocytosis markers, and complete blood count improved significantly, with TB 94.7 μmol/L, DB 76.3 μmol/L, ferritin 4451 ng/mL, sCD25–1090 U/mL, Hb 79 g/L, and platelet and WBC count normalized. The patient was discharged on ruxolitinib 5 mg qd and dexamethasone 3 mg qd. Ruxolitinib was continued for 1 month and then discontinued, while dexamethasone was gradually tapered and stopped. Post-discharge surveillance has been maintained at monthly intervals, with sustained afebrile status and normalized hepatic function to date.

### Case 3

A 41-year-old man was admitted on March 3, 2026, with a 1-month history of dizziness, fatigue, and recurrent fever. One month prior, the patient developed dizziness, fatigue, and fever (maximum 39.5 °C). He presented to a local hospital and was treated with Tibetan medicine (not specified), after which hepatic dysfunction developed, characterized predominantly by elevated liver enzymes. Following hepatoprotective therapy, liver enzymes improved markedly; however, bilirubin progressively increased, fever persisted, and cytopenia developed. The patient was then transferred to our hospital. On admission, the patient was remarkably jaundiced and had marked edema of the lower extremities and scrotum. The remainder of the examination was unremarkable. Laboratory tests revealed: hemoglobin 80 g/L, platelet count 13 × 10^9^/L, white blood cell count 0.22 × 10^9^/L, absolute neutrophil count 0.02 × 10^9^/L; PT 24.1 seconds, INR 2.08, APTT 76.0 seconds, TT 22.7 seconds, fibrinogen 1.50 g/L; TB 308.5 μmol/L, DB 229 μmol/L, total bile acids 187.7 μmol/L, ALT 309 U/L, AST 255 U/L, alkaline phosphatase 146 U/L, γ-glutamyl transferase 103 U/L, total protein 36.6 g/L, albumin 27.6 g/L, triglycerides 2.38 mmol/L, LDH 367 U/L; ferritin 40,606 ng/mL; sCD25 9,987 U/mL; procalcitonin (PCT) 5.780 ng/mL. Blood cultures grew *Streptococcus mitis* oralis group, and sputum cultures yielded *Candida albicans*. Chest CT scan revealed increased and blurred lung markings, with scattered patchy opacities, linear opacities, and areas of consolidation in both lungs, accompanied by small bilateral pleural effusions, consistent with pulmonary infection. Abdominal CT demonstrated hepatosplenomegaly, with more prominent hepatic enlargement. This patient presented with fever persisting over one week, pancytopenia, markedly elevated ferritin, sCD25 levels and hemophagocytosis revealed in bone marrow, establishing the diagnosis of HLH. Based on the clinical presentations, laboratory findings, and chest CT findings, pulmonary fungal infection and bloodstream bacterial infection were diagnosed. Therefore, empirical therapy with antibiotics and antifungal was initiated. Given the patient’s severe neutropenia and concurrent polymicrobial infections, granulocyte colony-stimulating factor (G-CSF) was administered for leukocyte recovery. For HLH control, following detailed counseling with the patient and family, and with written informed consent secured, treatment was initiated with ruxolitinib 5 mg bid plus daily intravenous dexamethasone 10 mg. Meanwhile, ademetionine butanedisulfonate and polyene phosphatidylcholine were used as adjunctive therapy. Furthermore, an extensive evaluation was undertaken to determine the underlying etiology of HLH. Bone marrow examination revealed hemophagocytosis, otherwise unremarkable; PET/CT was performed, revealing hypermetabolic infiltrative changes in the lungs consistent with infection; the remainder was otherwise unremarkable. HLH-associated mutations screening revealed a heterozygous PLCG2 mutation (c.-8G>A). The patient was advised to undergo a liver biopsy; however, he refused the procedure. After two weeks of treatment, the patient’s edema markedly resolved, liver function significantly improved, fever subsided, and hemophagocytic markers showed substantial amelioration. Follow-up chest CT demonstrated marked improvement. After 14 days of treatment, liver function was re-evaluated and showed TB 73.4 μmol/L, DB 55.7 μmol/L, ALT 110 U/L, and AST 48 U/L. Complete blood count showed: Hb 53 g/L, platelet count 57 × 10^9^/L, and WBC 11.42 × 10^9^/L. The patient was then discharged and was tapered to ruxolitinib 5 mg qd and dexamethasone 3 mg qd. Ruxolitinib was discontinued after one month, and dexamethasone was gradually tapered and stopped. Outpatient follow-up to date shows no fever and near-normalization of hepatic function and complete blood counts improved remarkably.

## Discussion

In this study, we describe three patients who initially presented with severe drug-induced liver injury (DILI) and were subsequently diagnosed with secondary hemophagocytic lymphohistiocytosis (HLH). The key clinical and laboratory features of these cases, summarized in **Table 1**, demonstrate that all patients fulfilled at least five of the eight HLH-2004 diagnostic criteria, including persistent fever, splenomegaly, cytopenia, hypofibrinogenemia or hypertriglyceridemia, elevated ferritin or sCD25, and/or hemophagocytosis.

**Table 1:** HLH diagnostic criteria fulfilled by three cases.

HLH-2004 diagnostic criteria		Case 1	Case 2	Case 3
Fever (>38.5 °C)		Yes (Maximum 40.0 °C; fever persisted >1 week)	Yes (Maximum 39.5 °C; fever persisted >1 week)	Yes (Maximum 38.5 °C; fever persisted >1 week)
Splenomegaly		Yes (on abdominal CT)	Yes (on	Yes (on abdominal CT)
			abdominal CT)	
Cytopenias (Affecting >= 2 lineages)	Hemoglobin (<90 g/L)	No	Yes (80 g/L)	Yes (71 g/L)
	Platelet (<100 × 109/L)	No	Yes (43 × 10^9^/L)	Yes (9 × 10^9^/L)
	Neutrophils (<1.0 × 10^9^/L)	No	Yes (0.77 × 10^9^/L)	Yes (0.02 × 10^9^/L)
Hypertriglyceridemia (Triglycerides >=3.0 mmol/L or >=265 mg/dL) or Hypofibrinogenemia (Fibrinogen <=1.5 g/L)		No	Yes (Triglycerides 3.13 mmol/L)	No
		Yes (Fibrinogen 0.78 g/L)	Yes (Fibrinogen 0.80 g/L)	No
Hemophagocytosis (bone marrow, spleen, or lymph nodes)		Yes (bone marrow)	No	Yes (bone marrow)
Ferritin (>=500 ng/mL)		No	Yes (45,174 ng/mL)	Yes (40,606 ng/mL)
sCD25 (>=2,400 U/mL)		Yes (2971 U/mL)	Yes (5,182 U/mL)	Yes (9,987 U/mL)
Decreased or absent natural killer (NK) cell activity		NA	NA	NA

**Figure 2:**
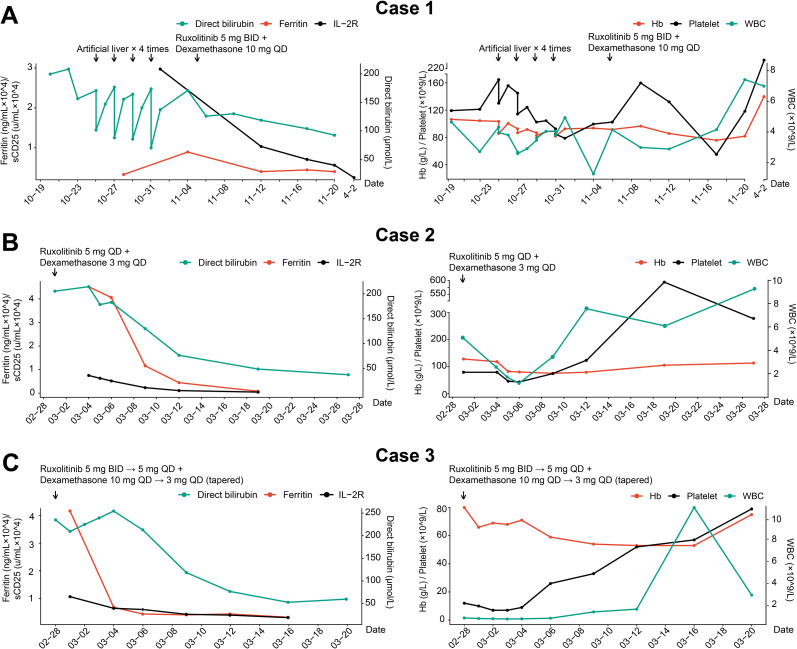
Changes in laboratory parameters following therapy of three cases. **(A–C)** Correspond to Case 1, Case 2, and Case 3, respectively. Laboratory markers of liver function, systemic inflammation, and hematologic status are shown over time. Following initiation of ruxolitinib-based therapy, inflammatory markers declined rapidly, accompanied by progressive improvement in liver function. Arrows indicate key treatment time points, including initiation of ruxolitinib and artificial liver support where applicable. Hb, hemoglobin; WBC, white blood cell; sCD25, soluble interleukin-2 receptor.

Notably, despite differences in initial presentation, all three patients exhibited a shared clinical trajectory, characterized by predominant hepatic involvement, rapidly progressive hyperbilirubinemia, and delayed recognition of HLH due to an initial diagnosis of DILI or liver failure. Treatment with low-dose ruxolitinib combined with corticosteroids was associated with rapid clinical and biochemical improvement. While all patients also received concomitant therapies—including artificial liver support, hepatoprotective agents, antimicrobial therapy, and G-CSF—the contribution of ruxolitinib with corticosteroids alone cannot be precisely determined. Nevertheless, as shown in the treatment timeline, bilirubin temporarily decreased following each session of artificial liver therapy but rebounded the next day. Similarly, prior hepatoprotective therapy at local hospitals produced minimal or transient improvement, with liver function worsening in some cases. Progressive and sustained declines in bilirubin and inflammatory markers were observed following initiation of ruxolitinib combined with corticosteroids, indicating that this combination likely played a major role in the clinical improvement observed in these patients (**Figure 2**).

### Drug-induced immune dysregulation as an underrecognized trigger of HLH

Drug-induced hypersensitivity syndrome (DIHS), also referred to as drug reaction with eosinophilia and systemic symptoms (DRESS), has increasingly been recognized as a trigger for secondary HLH (7, 8). Both conditions share overlapping clinical and immunological features, including fever, rash, cytopenia, and multiorgan involvement, suggesting a common spectrum of immune dysregulation (9). A recent review identified 23 reported cases of HLH-DRESS overlap, in which fever, hepatic involvement, and hyperferritinemia were present in most patients, and mortality reached approximately 24% (10, 11), highlighting the severity of this condition. Individual case reports have further illustrated that drug-induced HLH may present with prominent hepatic dysfunction and even mimic acute liver failure, thereby contributing to delayed recognition. Collectively, these findings suggest that drug-induced immune dysregulation, particularly in the context of DILI or DIHS, is an important but likely underrecognized trigger for HLH.

### Distinctive features of the present case series

Compared with previously reported cases of DIHS- or DRESS-associated HLH, our series demonstrates several distinct features. First, all three patients presented with severe hepatic dysfunction, marked hyperbilirubinemia, and clinical manifestations mimicking acute liver failure, whereas prior reports more commonly emphasized systemic hypersensitivity features such as rash and lymphadenopathy. Second, although most published cases were treated with corticosteroids, intravenous immunoglobulin, and in some cases etoposide, all three patients in our series were successfully treated with ruxolitinib combined with corticosteroids, with rapid clinical and biochemical improvement and without apparent hepatotoxicity. Third, variants in immune-regulatory genes, including NLRP3 and DOCK8, were identified in our patients but have rarely been described in earlier report. The identified heterozygous variants in NLRP3, DOCK8, MYO5A, and PLCG2 are considered VUS based on ACMG/AMP criteria and are not rare in East Asian populations. These variants may confer a subtle predisposition to secondary HLH in the presence of extrinsic triggers, but their pathogenicity remains unproven and hypothesis-generating. Functional studies are required to assess their role in disease susceptibility. Taken together, our findings broaden the clinical spectrum of drug-associated HLH and support ruxolitinib-based therapy as a potential option in patients with severe hepatic impairment (12). A summary of previously reported cases is presented in **Table 2**.

**Table 2:** Summary of published cases of DIHS/DRESS-associated HLH.

Study	Number	Population	Causative drug	Symptoms/HLH features	Hepatic involvement (including VBDS/liver failure)	Treatment
Gupta et al. (13),	1	Pediatric male	Phenytoin (antiepileptic)	DRESS, HLH, EBV reactivation	Severe hepatocellular injury (no VBDS or liver failure reported)	Steroids + etoposide
Hayashi et al. (14),	1	Adult female	Dietary supplement	Cytopenia, hyperferritinemia, hemphagocytosis	Acute liver failure with severe hepatocellular injury	Steroids + plasma exchange
Lemmens et al. (15),	1	Adult female	Boswellia serrata (herbal supplement)	Fever, rash, eosinophilia, cytopenia, HHV-6 positivity	Cholestatic hepatitis with VBDS (ductopenia)	Corticosteroids + etoposide + UDCA
Li et al. (16),	1	Adult female	Amoxicillin-clavulanate (antibiotic)	Fever, rash, cytopenia, hyperferritinemia, EBV reactivation	Severe cholestatic injury with VBDS progressed to liver failure	Steroids + IVIG + immunosuppressants
Liu et al. (17),	2	Adults (allopurinol)	Allopurinol	Fever, rash, eosinophilia, HLH in 2 cases	Liver injury with cases of acute liver failure (VBDS not reported)	Steroids ± IVIG
Pope et al. (18),	1	Pediatric	Amoxicillin-clavulanate + TMP-SMX (antibiotics)	Fever, rash, pancytopenia, multiorgan dysfunction	Mild hepatocellular injury (transaminitis only)	IVIG + steroids + anakinra
Xia et al. (19),	1	Adolescent female	Piperacillin-tazobactam (antibiotic)	Fever, pancytopenia, hyperferritinemia, mimicking infection (↑PCT)	Mild–moderate hepatocellular injury without liver failure	Drug withdrawal ± supportive care
Yang et al. (11),	23	Mostly adults	Antibiotics, antiepileptics, sulfasalazine (various)	Fever, rash, cytopenia, hyperferritinemia, multiorgan involvement	Hepatic involvement is common (elevated transaminases in 91%); severe liver failure/VBDS is not systematically reported	Corticosteroids ± IVIG/etoposide
Zhang et al. (20),	1	Elderly female	Carbamazepine (antiepileptic)	Fever, rash, cytopenia, hyperferritinemia	Severe cholestatic liver injury with VBDS (bilirubin >600 μmol/L)	Methylprednisolone + plasmapheresis + IVIG
Zhou et al. (21),	1	Adult female	Lamotrigine (antiepileptic)	Fever, cytopenia, hyperferritinemia, multiorgan involvement	Mild–moderate hepatocellular injury (transaminitis)	Steroids + etoposide

### A mechanistic link between hepatocellular injury and systemic hyperinflammation

The pathophysiological link between DILI and HLH is likely mediated by excessive immune activation triggered by hepatocellular injury. Damage to hepatocytes may lead to the release of damage-associated molecular patterns (DAMPs), which activate innate immune pathways and promote macrophage and T-cell activation (22). This response may then be amplified by dysregulated cytokine signaling, ultimately resulting in the hyperinflammatory state characteristic of HLH (1). In the setting of drug-induced hypersensitivity, this process may be further exacerbated by drug-specific T-cell activation and impaired cytotoxic function, leading to persistent antigenic stimulation and uncontrolled macrophage activation. Liver involvement itself may be central to this process, as the liver is an immunologically active organ enriched with resident macrophages that can rapidly propagate systemic inflammation once activated (23, 24). Our findings further suggest that genetic predisposition may lower the threshold for this transition, as variants in NLRP3 and DOCK8 may facilitate inflammasome activation and impair immune homeostasis, thereby promoting progression from localized liver injury to systemic hyperinflammation (25). However, the proposed involvement of T cells and Kupffer cells in DILI-associated HLH is a hypothesis supported by clinical and genetic observations rather than direct immunohistochemical or immunofluorescence evidence. Future studies employing tissue-based or single-cell analyses are needed to confirm the cellular mediators of hyperinflammation in this context.

### Therapeutic rationale for JAK-STAT inhibition in hepatic-predominant HLH

**Figure 3:**
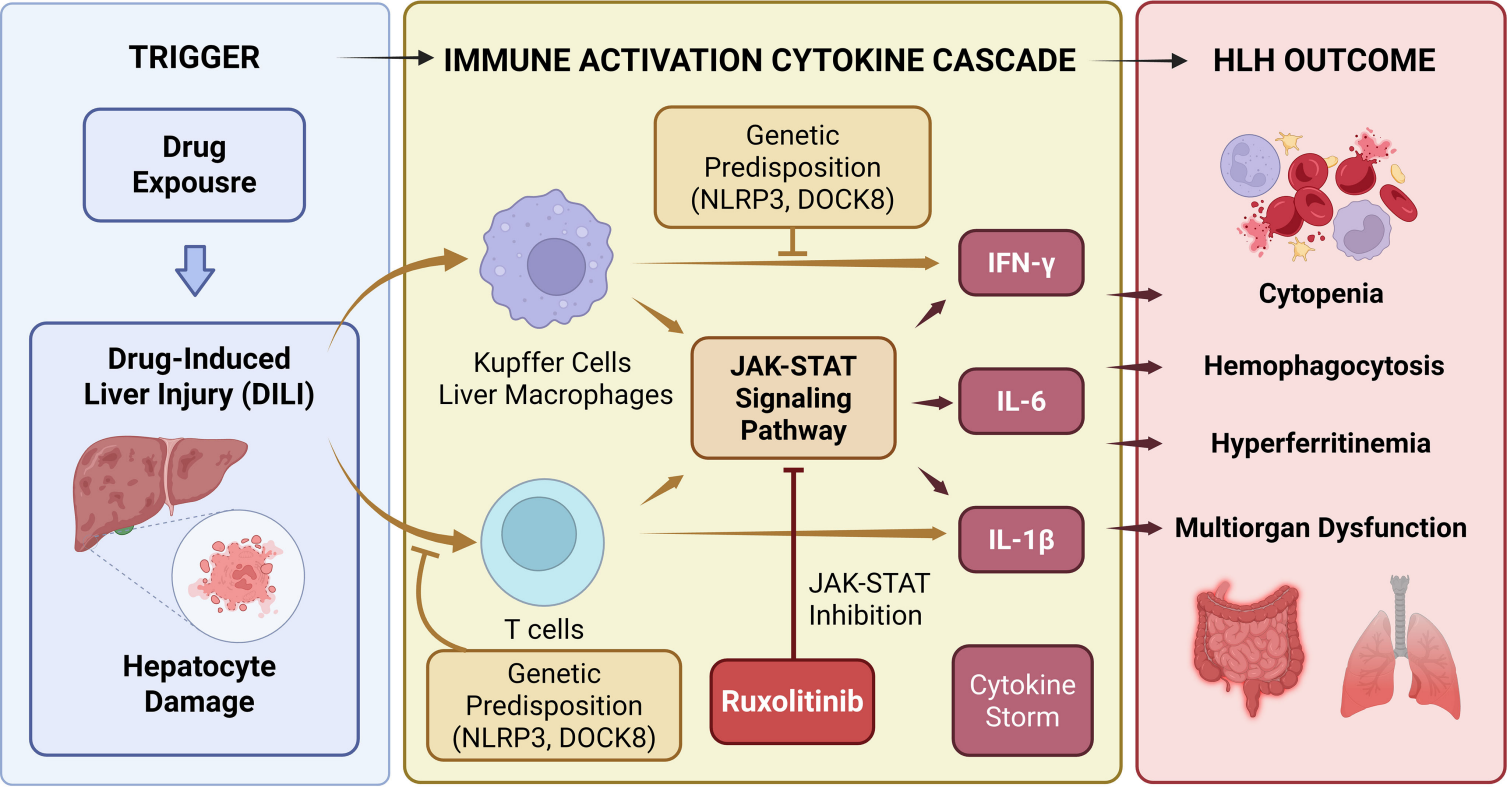
Proposed mechanism of DILI-associated HLH and therapeutic role of ruxolitinib. Drug exposure leads to hepatocellular injury, resulting in the release of damage-associated molecular patterns, which activate innate immune responses, including Kupffer cells and other macrophages. This activation promotes T-cell-mediated immune responses and triggers a cytokine cascade characterized by increased levels of interferon-γ, interleukin-6, and other proinflammatory mediators. In genetically susceptible individuals (e.g., NLRP3 or DOCK8 variants), dysregulated immune responses may further amplify cytokine signaling, leading to uncontrolled macrophage activation and the development of HLH. Ruxolitinib inhibits the JAK-STAT signaling pathway downstream of multiple cytokine receptors, thereby attenuating the cytokine storm and controlling hyperinflammation, while potentially avoiding additional hepatic injury associated with cytotoxic therapies.

The observed efficacy of ruxolitinib in our patients may be explained by its inhibition of the JAK-STAT signaling pathway, which plays a central role in cytokine-mediated immune activation (6). By suppressing downstream signaling of key inflammatory mediators, including interferon-γ and interleukin-6, ruxolitinib may attenuate the cytokine storm without directly aggravating hepatic injury (26, 27). This mechanism is particularly relevant in patients with severe liver dysfunction, in whom etoposide-based regimens may be difficult to tolerate because of concerns regarding toxicity (28). This mechanistic framework may also explain why patients with predominant hepatic involvement are particularly prone to rapid deterioration and why early targeted immunomodulation may be especially important in this subgroup (**Figure 3**).

### Clinical implications for recognition and treatment selection

From a clinical perspective, our findings underscore the importance of early recognition of HLH in patients with suspected DILI. HLH should be considered in patients with persistent high-grade fever, progressive cytopenia, coagulopathy, and markedly elevated inflammatory markers such as ferritin and sCD25, particularly when these abnormalities cannot be fully explained by infection or fail to improve with conventional anti-infective or hepatoprotective therapy (29). Timely differentiation between isolated DILI and DILI-associated HLH is critical, as delayed diagnosis may lead to rapid progression and poor outcomes. In such cases, early evaluation with bone marrow examination and assessment using HLH-2004 criteria or HScore should be strongly considered (30). Although etoposide-based regimens remain standard therapy for HLH, our cases suggest that ruxolitinib combined with corticosteroids may represent an effective and well-tolerated alternative in patients with predominant liver involvement, high inflammatory burden, or contraindications to cytotoxic therapy (31). Overall, our findings highlight that DILI-associated HLH is a distinct and clinically recognizable entity with significant therapeutic potential, especially when early diagnosis enables the timely initiation of targeted immunomodulatory therapy.

In our case series, short-course, low-dose ruxolitinib combined with corticosteroids led to rapid clinical and biochemical remission without significant treatment-related complications. Transient declines in blood counts were self-limited and recovered without intervention. Nevertheless, these observations should be interpreted in the context of certain limitations: The specific drugs responsible for liver injury could not be fully identified in these cases. Given the use of multiple concomitant therapies, the observed clinical improvement cannot be attributed solely to ruxolitinib combined with corticosteroids, and future prospective studies are needed to validate its efficacy and support broader application of this treatment approach.

## Conclusion

In conclusion, our case series highlights drug-induced liver injury as an important but underrecognized trigger of HLH, particularly in patients presenting with prominent hepatic dysfunction. The overlap between DILI and HLH poses a significant diagnostic challenge, as clinical manifestations may mimic acute liver failure and delay appropriate management. Our findings suggest that ruxolitinib combined with corticosteroids may represent an effective and well-tolerated therapeutic strategy in patients with severe hepatic involvement, especially when conventional etoposide-based regimens are relatively contraindicated. Early recognition and prompt initiation of targeted immunomodulatory therapy may be critical to improving outcomes in this potentially life-threatening but treatable condition.

## Data Availability

The original contributions presented in the study are included in the article/supplementary material. Further inquiries can be directed to the corresponding authors.
